# Modelling hepatocellular carcinoma microenvironment phenotype to evaluate drug efficacy

**DOI:** 10.1038/s41598-024-84304-4

**Published:** 2025-01-07

**Authors:** Sara Cherradi, Salomé Roux, Marie Dupuy, Séverine Tabone-Eglinger, Edouard Tuaillon, Marianne Ziol, Eric Assenat, Hong Tuan Duong

**Affiliations:** 1 PredictCan Biotechnologies SAS, Biopôle Euromédecine, Grabels, France; 2 Service d’Oncologie Médicale, Hôpital Saint Eloi, Centre Hospitalier Universitaire de Montpellier, Montpellier, France; 3 Centre de Ressources Biologiques (CRB), Centre Hospitalier Universitaire de Montpellier, Montpellier, France; 4 Plateforme de Gestion des Echantillons Biologiques, Centre Léon Bérard, Cancer Research Center of Lyon, Université de Lyon, Université Claude Bernard Lyon 1, INSERM 1052, CNRS 5286, Lyon, France; 5 Centre de Ressources Biologiques du Groupe hospitalier Paris Seine Saint-Denis, Paris, France

**Keywords:** Patient-centric spheroid model, Hepatocellular carcinoma, Targeted therapy, Tyrosine kinase inhibitors, Prediction of clinical outcomes, Cancer models, Hepatocellular carcinoma, Tumour heterogeneity

## Abstract

Hepatocellular carcinoma (HCC) is the third most common cause of cancer-related death worldwide. Treating HCC is challenging because of the poor drug effectiveness and the lack of tools to predict patient responses. To resolve these issues, we established a patient-centric spheroid model using HepG2, TWNT-1, and THP-1 co-culture, that mimics HCC phenotype. We developed a target-independent cell killing (TICK) exclusion strategy to monitor the therapeutic response. We demonstrated that our model reproduced the Barcelona Clinic Liver Cancer (BCLC) molecular classification, displayed known alterations of epigenetic players, and responded to tyrosine kinase inhibitors (TKIs) such as sorafenib, cabozantinib, and lenvatinib in a patient-dependent manner. Importantly, we reported for the first time that our model correctly predicted 34 clinical outcomes to TKIs out of 37 case studies on 32 HCC patients confirming that patient-centric spheroids, combined with our TICK exclusion strategy, are valuable models for drug discovery and opening a near perspective to personalized care.

## Introduction

Although hepatocellular carcinoma (HCC) is the third most common cause of cancer-related death and the leading cause of death among cirrhotic patients, only few treatment options are available^1^. Moreover, HCC is often diagnosed at an advanced stage when curative approach such as liver resection is no longer feasible^2^. Thus, patients could benefit only from systemic therapies including immunotherapies, the current standard of first line treatment, or tyrosine kinase inhibitors (TKIs) such as sorafenib, cabozantinib, lenvatinib, or regorafenib^3^, mainly in subsequent lines. Unfortunately, TKIs’ therapeutic efficacy is generally poor, presumably because of the tumor heterogeneity which is a pivotal factor in drug resistance, and their apparent toxicity is high^4^. Thus, there is an urgent need for effective medical therapy. To that end, experimental models that could mimic tumor heterogeneity are key components of anti-cancer drug development. Hepatoma cell lines such as Huh7, HepG2, or Hep3B, HCC organoids, HCC PDX mouse, and HCC patient-derived spheroids are widely used to evaluate the efficacy of anti-cancer compounds and for drug discovery. However, results are unsatisfactory because they cannot reproduce the clinical outcomes of treated patients. Llovet and colleagues have reported in a clinical trial that 43% of treated patients responded to sorafenib^5^. However, experimental studies showed that most hepatoma cell lines, 78% of HCC organoids, and 64% of HCC patient-derived spheroids, were sensitive to sorafenib contradicting the proportion of responses observed in the clinic^6–9^.

In clinical practice there is no decision-making marker to predict therapeutic response and safety. One strategy to predict the response is measuring biomarkers to know whether a gene mutation/expression is linked to the response to a drug for a given patient. However, this method does not allow testing at once several drugs on the same patient. The second approach relies on HCC organoids and patient-derived xenograft models that are unsatisfactory because their predictivity of clinical outcomes is poor. Indeed, HCC organoids that retained mutations found in the original tissues, failed to predict the clinical outcome to therapeutics suggesting that mutations are less important than the tumor phenotype in regard to cancer cells sensitivity to treatments^10^.

The tumor microenvironment (TME) is complex as it contains diverse cell types interacting between each other to create a favorable condition for tumor cells survival and growth^11^. That microenvironment is also important for the treatment response of the tumor as it modulates the sensitivity of cancer cells to therapeutics^12^. Interestingly, from the organoids data it appears that preserving the original cell population and mutations of the tumor is clearly not sufficient to predict the malignant cells behavior in response to anticancer drugs, suggesting that other components of the tumor microenvironment, for instance intra- and inter-organ communicating molecules, are required in the cell culture system to accurately reproduce the tumor phenotype.

To resolve that issue, we have developed an HCC patient-centric model using HCC patient’s serum to educate cell line-based spheroids mimicking the tumor phenotype. This proprietary cell educating technology was validated elsewhere^13–15^. These spheroids are co-cultures of HepG2 and THP-1 lines from ATCC, and TWNT-1 line from Glow Biologics.

## Results

### HCC patient-derived serum educated spheroids reproduce the BCLC molecular classification

**Fig. 1:**
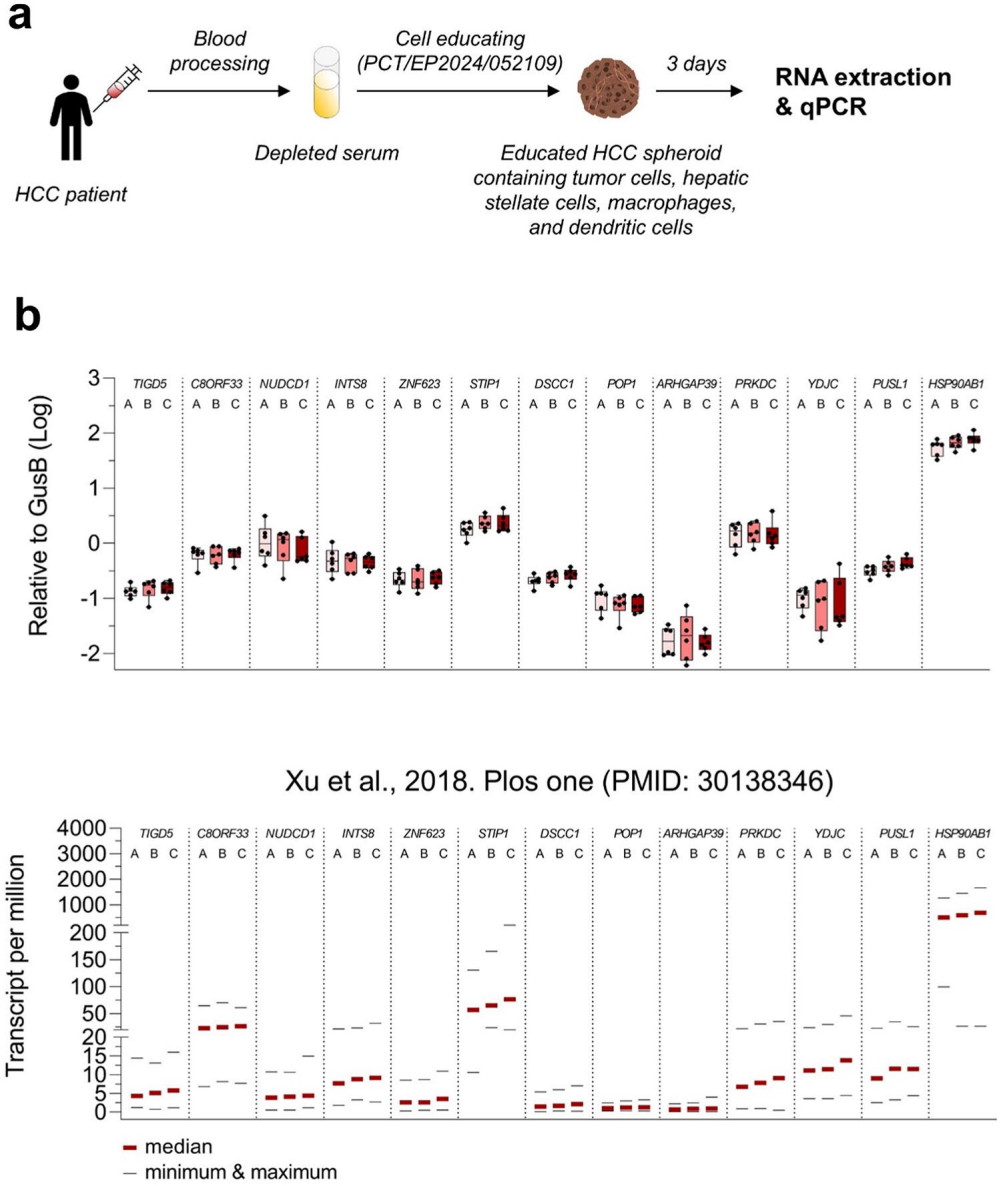
Staging of HCC patient-derived serum educated spheroids based on the molecular signature described by Xu and colleagues. (**a**) Workflow showing the generation of PDSES using depleted serum from HCC patients with known BCLC staging. (**b**) Upper graph. Quantitative PCR analysis of the expression of the 13 hub genes in PDSES. Results are reported as Log expression relative to GusB gene. Depleted serum from 6 HCC patients with BCLC-A, BCLC-B, and BCLC-C were used to generate the spheroids. After 3 days of culture, total RNA was prepared for qPCR analysis of the 13 hub genes. Lower graph. Expression of 13 hub genes that are correlated with the overall survival in various stages. Results were extracted from the manuscript of Xu et al., 2018. Plos one (PMID: 30138346) and are shown as transcript per million. Thick lines show the median and thing lines depict the maximum and the minimum values.

The response to TKIs treatment in HCC depends on the Barcelona Clinic Liver Cancer (BCLC) classification, a staging system that is based on tumor characteristics, liver function, and patient performance status^16^. Recently, Xu and colleagues have identified a set of 13 hub-genes that correlate with the BCLC staging system and the prognosis of patients with HCC^17^ (Fig. 1b). In order to verify whether these hub-genes are also expressed in HCC patient-derived serum educated spheroids (PDSES) and do correlate with BCLC staging, we generated PDSES using blood serum from 18 HCC patients with known BCLC staging (Fig. 1a). Hub-genes were measured by quantitative PCR three days after the cell educating step. Although we have only a small sample size (18 HCC patients) in our study compared to the study performed by Xu and colleagues (249 HCC patients), we found a similar trend of expression of the 13 hub-genes (Fig. 1b). Moreover, among the 13 hub-genes, 5 genes (*TIGD5*, *STIP1*, *DSCC1*, *PUSL1*, and *HSP90AB1*) clearly showed an increased expression that matches with the BCLC staging (Fig. 1b).

### HCC patient-dependent expression of epigenetic players in HCC patient-derived serum educated spheroids

**Fig. 2:**
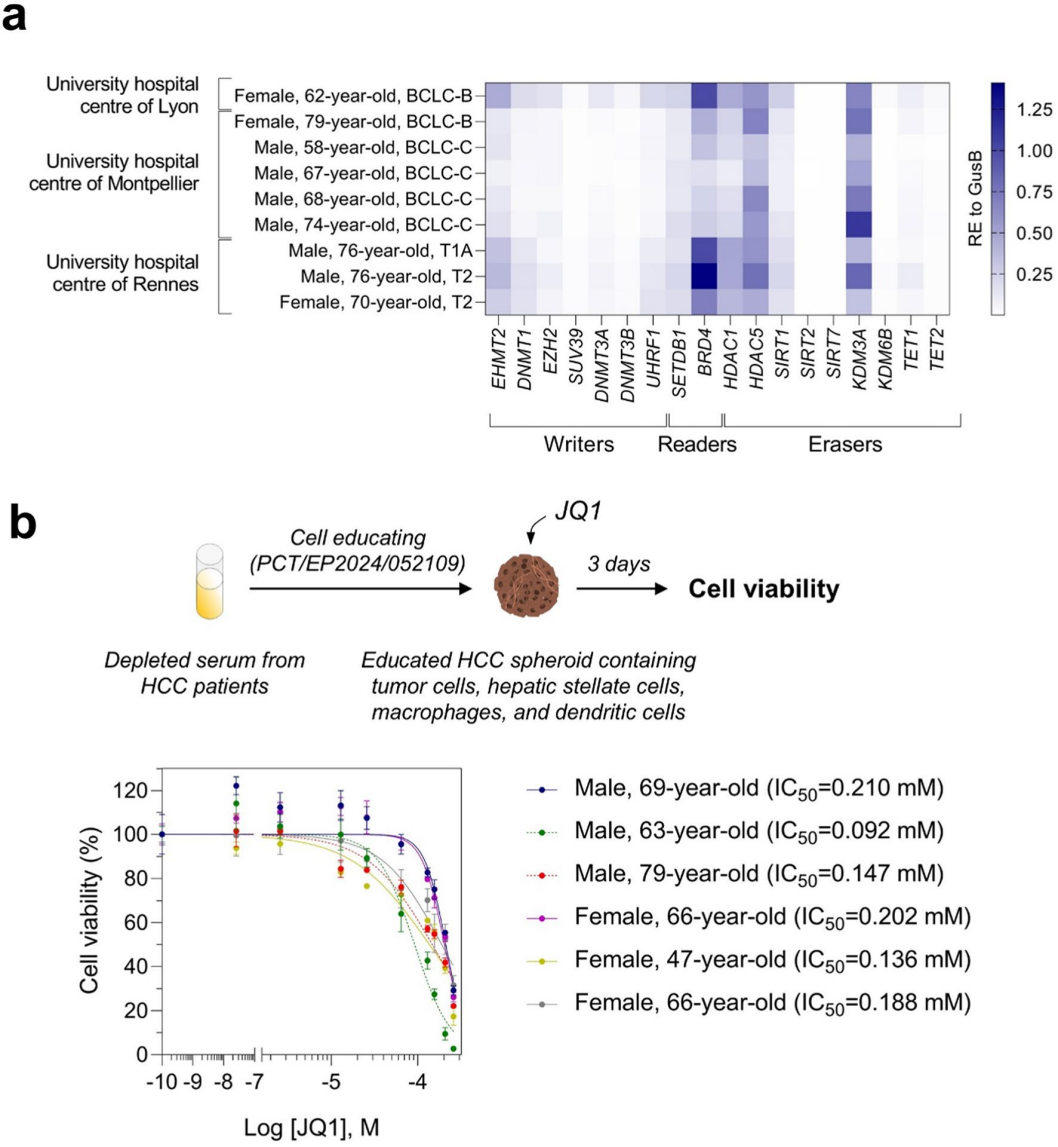
HCC patient-derived serum educated spheroids display an interindividual diversity of alteration of epigenetic players and sensitivity to the selective inhibitor of BRD4. (**a**) Blood sera from multiple centres were used to generate PDSES. The expression of epigenetic players including the writers, readers, and erasers was analyzed by quantitative PCR. Results are expressed as relative to GusB gene. (**b**) PDSES were generated with blood sera from 3 males and 3 females and then treated with JQ1. Cell viability was measured 3 days post-treatment. An inhibitory dose-response curve fit with constraints (top=100; bottom=0) was drawn for each patient.

The epigenetic machinery that regulates gene expression through chromatin remodelers including writers, readers, and erasers, is altered in HCC^18^. For instance, the expression of writers such as EHMT2, readers such as BRD4, or erasers such as HDAC5 is high in tumor tissues^19–21^. We analyzed the expression of a panel of epigenetic players in PDSES and found that among the epigenetic players, *EHMT2*, *BRD4*, *HDAC1*, *HDAC5*, and *KDM3A* were highly expressed in a patient-dependent manner (Fig. 2a). BRD4 is a histone modification reader and transcriptional regulator that is a target for treatment of HCC^21^. To confirm that hypothesis, we generated PDSES and then treated them with JQ1, a selective small molecule inhibitor that binds competitively to bromodomains. As expected, we found a patient-dependent cancer cells death in response to JQ1 treatment correlating with a heterogeneous expression of BRD4 in PDSES (Fig. 2b). Our data demonstrated that epigenetic players are altered in a patient-dependent manner in PDSES and that these spheroids are valuable models to test the efficacy of small molecule inhibitors such as JQ1.

### Analysis of the response to tyrosine kinase inhibitors by target-independent cell killing exclusion strategy

**Fig. 3:**
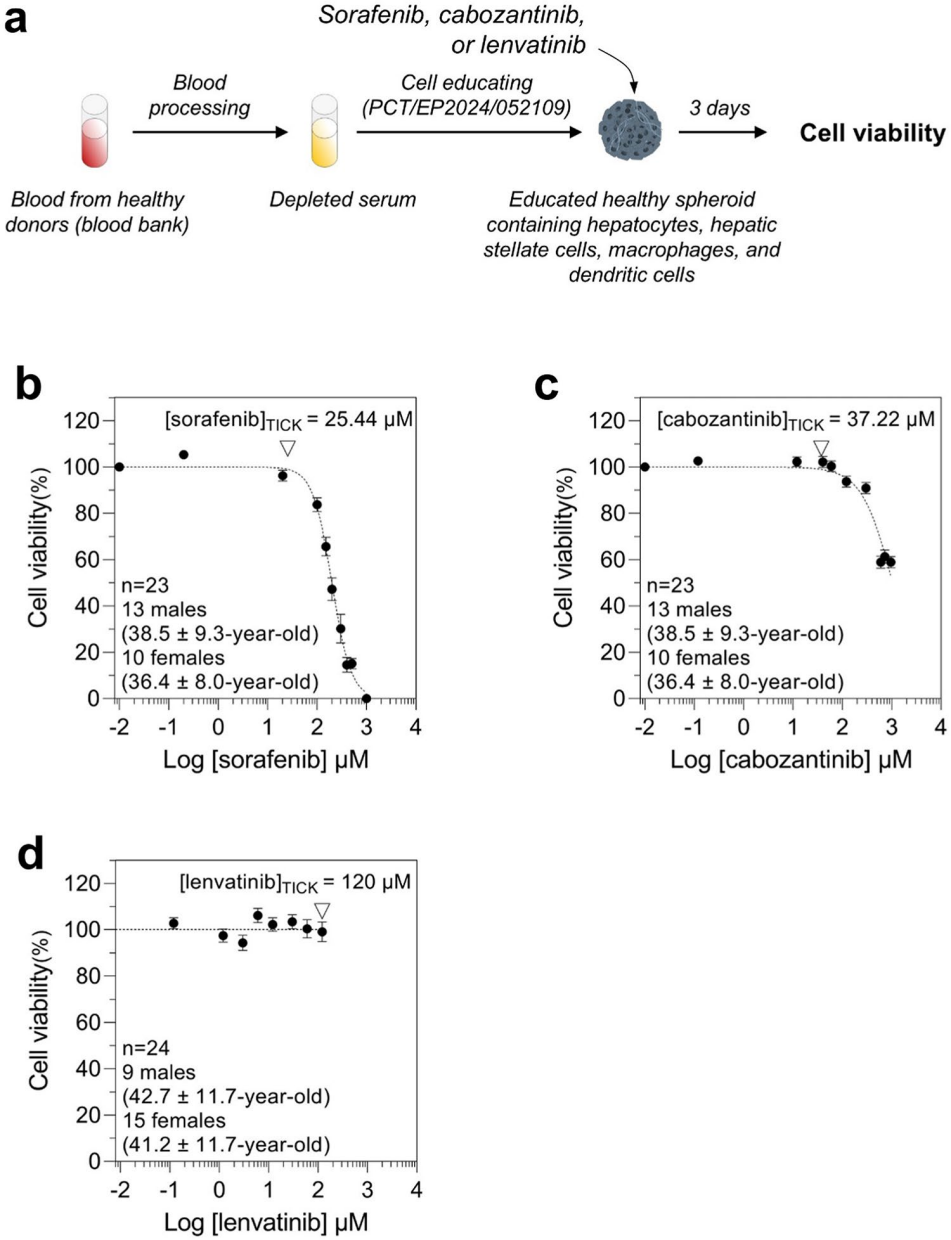
Determination of the target-independent cell killing (TICK) value for each TKI. (**a**) Individual-centric spheroids were generated using depleted blood sera from healthy individuals. Spheroids were then treated with sorafenib, cabozantinib, and lenvatinib. Cell viability was measured at day 3 post-treatment. For each TKI, an inhibitory dose-response curve fit with constraints (top = 100; bottom = 0) was drawn. Each condition was performed in triplicate for each individual. (**b**) Sorafenib and (**c**) cabozantinib curves show an average cell viability for each concentration of the cohort. TICK values were defined as the first percentage of cell death. (**d**) For lenvatinib, no cell death was observed up to 240 x C_max_. TICK value was then set to the maximum concentration of 120 µM.

Although chemotherapy is largely opted as therapeutic option, its use is somehow limited by lack of selectivity as it can also kill or damage normal cells. Therefore, for drug efficacy validation, it is crucial to distinguish the true anticancer effect from the death caused by the drug in a healthy condition. To do so, we determined the target-independent cell killing (TICK) value for each TKI using cohorts of spheroids derived from healthy individuals, called healthy individual-centric spheroids, generated with blood serum from healthy individuals (Fig. 3a). After the cell educating step, healthy individual-centric spheroids were treated with increasing doses of sorafenib, cabozantinib, or lenvatinib, and cell viability was measured. The TICK value for each drug is defined as the average first percentage of cell death of the treated cohort. We found that TICK values for sorafenib and cabozantinib were 25.44µM and 37.22µM, respectively (Fig. 3b and c). For lenvatinib, TICK value was set to the maximum value of 120µM as we did not measure any cell death at lower concentrations (Fig. 3d). Once determined, these TICK values will be used as cut-off values to measure the anticancer effect of corresponding drug.

**Fig. 4:**
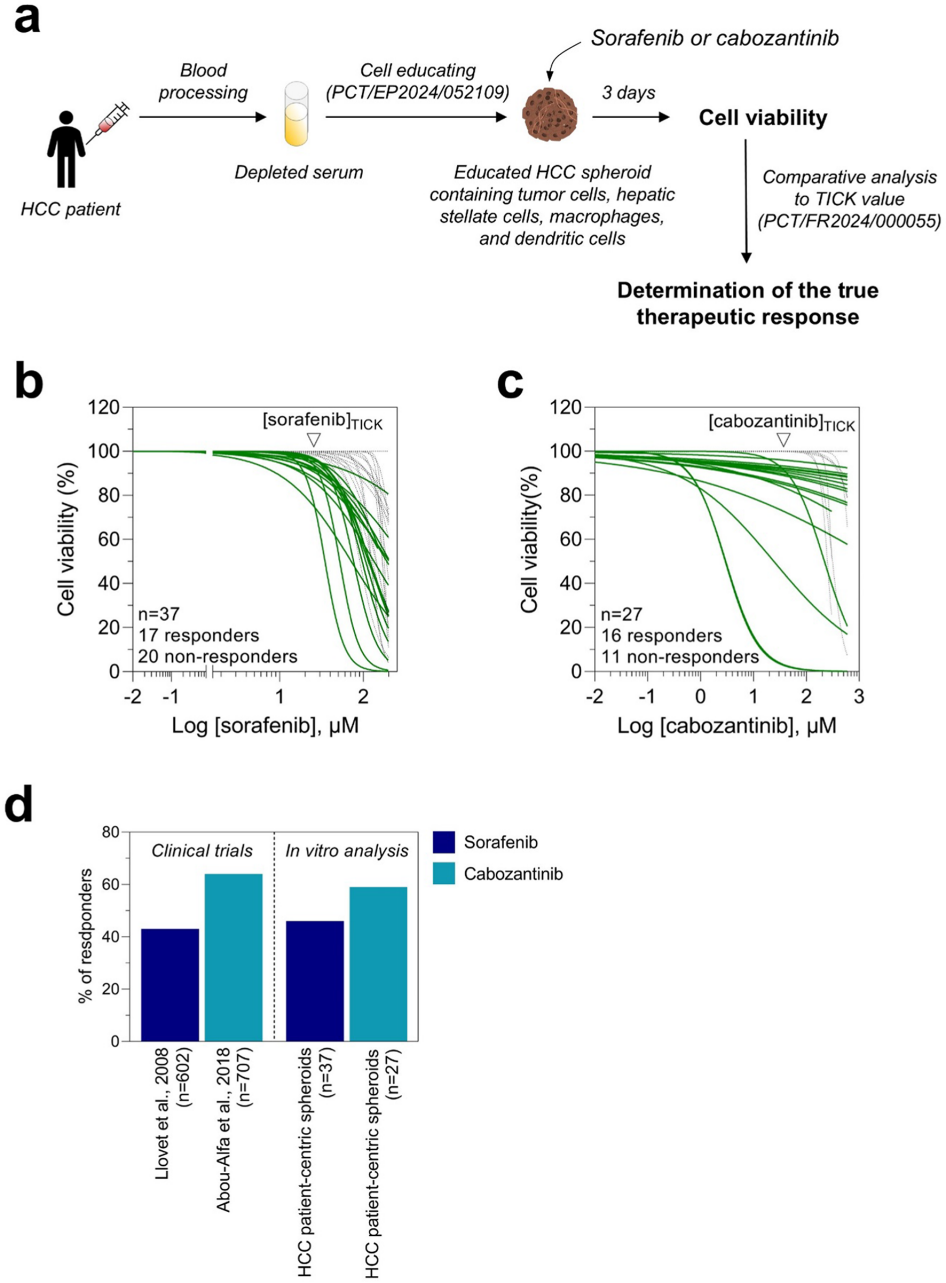
HCC patient-derived serum educated spheroids show a diversity in responses to sorafenib and cabozantinib, and proportionally matched to results from clinical trials. (**a**) PDSES were generated with depleted blood sera from HCC patients and then treated with sorafenib or cabozantinib. Cell viability was measured 3 days post-treatment. An inhibitory dose-response curve fit with constraints (top = 100; bottom = 0) was drawn for each HCC patient and the true efficacy response was determined based on the TICK values. (**b**) True efficacy of sorafenib on a cohort of 37 HCC patients. Based on the TICK exclusion strategy, 17 HCC patients out of 37 were responding to sorafenib (shown with green curves). (**c**) 16 HCC patients out of 27 treated with cabozantinib were responding (shown with green curves). (**d**) Comparative analysis of the proportion of responders between in vitro data and results from 2 clinical trials.

We analyzed the response to sorafenib and to cabozantinib in 2 cohorts of PDSES using the TICK exclusion strategy. Experimentally, PDSES were generated with blood serum from HCC patients, and we performed a dose-response to sorafenib and to cabozantinib (Fig. 4a). Based on non-linear fitting curves and TICK values of sorafenib and of cabozantinib, we found that PDSES from 17 HCC patients out of 37 (45.9%) were sensitive to sorafenib, and from 16 HCC patients out of 27 (59.3%) were responding to cabozantinib (Fig. 4b and c). Previously, 2 randomized, double-blind, phase 3 trials were performed^5,22^. In those studies, HCC patients were treated with sorafenib or with cabozantinib. The authors found that the disease control rate was 43% for sorafenib and 64% for cabozantinib. Interestingly, we showed that 45.9% and 59.3% of PDSES were responding to sorafenib and to cabozantinib, respectively, approaching the values obtained from clinical trials, despite the smaller sampler size in our study (Fig. 4d). Our data show that PDSES results are proportionally similar to clinical results suggesting that our model can be potentially used to assess individually the response to TKIs.

### Results obtained from HCC patient-derived serum educated spheroids matched with clinical outcomes of patients treated with TKIs

**Fig. 5:**
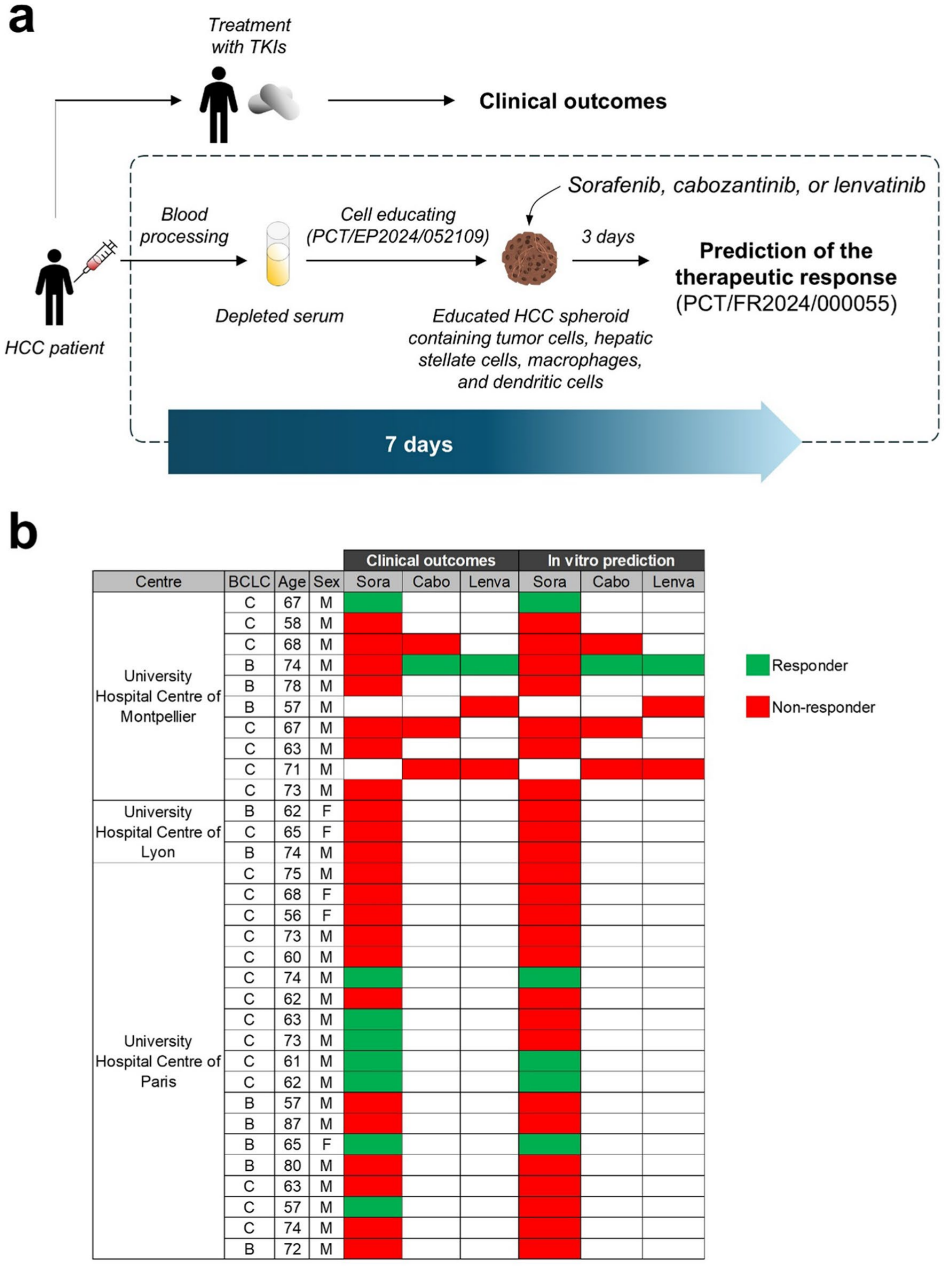
Evaluation of the performance of HCC patient-derived serum educated spheroids to reproduce the clinical outcomes to TKIs. (**a**) Blood sera from HCC patients were collected before the patient underwent a clinical treatment protocol. PDSES were generated and then treated with the same treatment as the one that he/she received in the clinic. A blind and unbiased in vitro analysis of the response to TKIs was performed. The true therapeutic response to TKIs was determined using the TICK exclusion strategy as described above. The full process from blood processing to final reports was 7 days. (**b**) Clinical outcomes and in vitro prediction responses to TKIs. PDSES from 29 HCC patients were generated and then treated with TKIs accordingly to the clinical treatment protocol. The true therapeutic efficacy was determined based on the TICK values for each TKI as described above.

To explore whether PDSES can reproduce clinical outcomes of HCC patients treated with TKIs such as sorafenib, cabozantinib, or lenvatinib, we performed 37 exploratory case studies on 32 HCC patients. Blood samples were collected from the University Hospital Centre of Montpellier, of Lyon, and of Paris, prior patients underwent a treatment protocol. Blood serum from each patient is prepared and then stored at -80 °C until use. PDSES were generated using blood serum from HCC patients and then treated accordingly to the clinical treatment protocol. We used TICK values from each TKI, as cut-off, to evaluate the treatment response in our in vitro system (Fig. 5a). From the 37 exploratory case studies, 8 positive clinical responses to sorafenib, 1 to cabozantinib, and 1 to lenvatinib were observed. Using our PDSES, we found that 5 positive in vitro responses to sorafenib matched with the clinical outcomes. Both cabozantinib and lenvatinib in vitro responses matched with clinical results. Overall, out of the 37 conditions tested, only 3 false negative in vitro results were observed with sorafenib (Fig. 5b).

We also noticed that there is a discrepancy in the percentage of responders to sorafenib between our exploratory case study cohort (Fig. 5b: 26.7%) and the independent test cohort (Fig. 4b: 45.9%). Further analysis did not reveal any differences in terms of number of males/females and age average between the 2 cohorts (exploratory case study cohort in Fig. 5b: 83% males, 17% females, age average 67.6 ± 7.6-year-old; test cohort in Fig. 4b: 86% males, 14% females, age average 67.7 ± 6.3-year-old). However, we observed that only patients with BCLC-B (10 out of 30 treated with sorafenib) and BCLC-C (22 out of 30 treated with sorafenib) were found in the exploratory case study cohort while the test cohort included patients with BCLC-A (5 out of 37), BCLC-B (6 out of 37), BCLC-C (6 out of 37), T1B (8 out of 37), T2 (10 out of 37), and staging of 2 patients were not determined. It is known that the response to sorafenib depends on the stage and less advanced stage are generally more responsive to treatments like sorafenib^23,24^. This could explain the observed discrepancy. Altogether, our proof-of-concept study confirms that our system is a reliable laboratory-developed test (LDT) and encourages further investigations on larger cohorts of HCC patients under the guidance of the regulators.

## Discussion

In the present work, we demonstrated that PDSES can reproduce the molecular classification of BCLC staging described by Xu and colleagues^17^. We confirmed known alterations of expression of epigenetic modifiers such as BRD4, HDAC5, or KDM3A. We showed that JQ1, a potent inhibitor of BRD4, dose-dependently reduced tumor cells viability in PDSES. We found an interindividual heterogeneity of the responses to therapeutics in PDSES. We showed that 45.9% and 59.3% of treated PDSES were sensitive to sorafenib and to cabozantinib, respectively, matching with the proportion of the responses observed in clinical trials. And finally, we demonstrated in case studies that PDSES accurately predicted clinical outcomes of HCC patients treated with TKIs. Our results demonstrate that our non-invasive PDSES are highly valuable preclinical models for drug candidates’ selection at discovery stage and opens perspectives for personalized medicine.

In vitro models that are used to analyze the efficacy of new therapeutic compounds outside the pathological context often fall short in replicating the true responses that occur in a living organism. This can lead to discrepancies between the data obtained from in vitro analysis and those from treated patients. There are several reasons why in vitro models might not fully capture clinical responses. Among those, the cellular environment in the body, including factors like blood flow, tissue composition, and cell-to-cell interactions, is difficult to reproduce in vitro. In the present work, we demonstrate that it is possible to mimic the cellular environment of each patient by bringing the blood sera at physiological proportion to a multicellular cell line-based system. Importantly, we show that our patient-centric spheroids can analyze the true therapeutic response mirroring the clinical outcomes. Attempts to use HCC organoids to analyze the response to sorafenib have been made previously. These HCC organoids retained cellular architectures and mutations found in the original tissues. However, the authors observed that all HCC organoids were responsive to sorafenib^9,25^ contradicting the clinical outcomes where only 43% of treated patients showed a disease control^5^. The interindividual variability in drug response has become an acceptable fact in modern medicine and pharmacogenomic is widely used to get information about how a patient will respond to a treatment. Although there is evidence that pharmacogenomic can contribute to improve the success rate in the clinic, its use remains challenging as a complex combination of multiple mutations is presumably involved in the sensitivity to certain drugs, because of genetic redundancy. Our data highlighted a fundamental question whether the tumor phenotype could play a more important role than genomic in drug response. Further analyses are required to better understand how much the tumor phenotype and of the genomic contribute to drug response.

HepG2 are well-differentiated cells with a high proliferation rate making that they are sensitive to sorafenib-induced apoptosis when cultured in standard 2D cell culture conditions^26,27^. That sensitivity to sorafenib was reported to reduce when these cells are cultured as mono-population in 3D^28^. Our system is a multi-population spheroid model generated with the cell educating technology to alter the properties of cell lines towards a healthy normal phenotype or to a cancer phenotype in a subject-dependent manner. Furthermore, our spheroids are cultured in a defined proprietary basal cell culture medium. Altogether, our specific cell culture condition causes a reduction of sensitivity of our spheroids to sorafenib as compared to standard HepG2 models explaining the shift of the IC_50_ observed in Fig. 3b.

The tumor microenvironment that composes of non-cancerous cells including hepatic stellate cells and immune cells, and components that are produced and released by cancer cells, plays a crucial role in the response of tumor cells to therapies. Lastly, there is accumulating evidence that the human microbiota plays a role in therapeutic response as well. Indeed, animal experiments have revealed that microbes from the gut can facilitate the initiation and the progression of some cancers including hepatocellular carcinoma^29,30^. Importantly, these experiments also showed that some microbes are responsible for a poor response after chemotherapy^31,32^. Microbes can impact the distant tumor in a contact-independent manner via the gut microbiota-derived peptidoglycan fragments from the cell wall, that enter the blood stream^33^. Host organs differentially take up circulating peptidoglycan fragments. In the liver, it has been shown experimentally that the maximal uptake was 1 h and then remained stable for at least 6 h^34^. These peptidoglycan fragments have an average size ranging from 30 μm to 200 μm^35^. These fragments are sensed for instance by PGLYRP2, a constitutively expressed and liver specific pattern recognition receptor, that is downregulated in HCC, modulating the antitumor immune response^36^. The HCC patient-derived serum educated spheroid system that we have developed contains processed blood serum at physiological amounts, and thus a patient-specific composition of gut microbiota-derived peptidoglycan fragments, that can modulate the sensitivity to TKIs in a patient-dependent manner. Further analyses are needed to understand the mechanism by which these gut-derived peptidoglycan fragments, that are present in blood serum, are sensed by liver tumor cells modulating their sensitivity to therapeutics.

At high doses, anticancer drugs do not discriminate cancer cells from healthy bystander cells^37^. Thus, it is important for drug efficacy evaluation to define a cut-off value from which one could say whether the killing effect is on cells with a cancerous phenotype, or it is on cells with a healthy phenotype. Of note, this cut-off value is specific to each drug as the killing effect on healthy cells does not occur at the same dose for every drug. The flexibility of the cell educating technology allows us to define that cut-off value, called TICK, using individual-centric spheroids generated with blood serum from healthy individuals for each TKI, improving the accuracy of drug efficacy analysis on PDSES. Applying such strategy, one could determine a TICK value for each therapeutic compound and thus, could propose a laboratory-developed test to predict the clinical response to a panel of approved anti-HCC drugs for a better selection of the optimal treatment for each patient. As most HCC patients will undergo treatment in second- or third-line treatment with TKIs, because of the low efficacy of immunotherapies, using PDSES, we can challenge with a panel of FDA-approved TKIs on the same patient to establish a chemogram and identify the best treatment option (Fig. 5b). Our LDT is a decision-making tool for clinicians to tailor patient treatment. Our results encourage to pursuit further investigations under the guidance of regulators such as the Federal Drug Administration (FDA) and the European Medicines Agency (EMA), through prospective studies on larger cohorts to confirm the accuracy of our model to predict the clinical outcomes to small molecule targeted anti-cancer drugs. Finally, to our best knowledge, this is the first study reporting a comparative analysis of in vitro responses to the clinical outcomes supporting the validity of using PDSES as models to accurately evaluate drug efficacy at early drug development stage.

We are aware of the limitations of our system. First, it does not contain primary cells from patients and therefore our model cannot be used to study the biology of HCC nor the role of the genomic in drug response as it does not retain original mutations found in the patients. Nevertheless, the systemic components (i.e. cytokines, hormones, growth factors, EV, …) from the blood serum (the composition and the magnitude of their expression is specific to each patient) can stimulate cell lines to mirror the primary cell phenotype. Our data showed a clear phenotype heterogeneity after the education step and our exploratory case studies confirmed that using blood sera from HCC patients on cell lines was sufficient to mimic HCC phenotype of each patient for TKIs efficacy evaluation. Second, the mechanism of cell education remains unclear. The properties of the cells modulate their sensitivity or their resistance to TKIs, and it is not dependent on a unique factor but rather on a combination of signaling pathways that are activated or repressed in cancer cells. For instance, it has been reported that the sensitivity to TKIs is determined by HER3, ERK1/2 and p53 phosphorylation^38^. Knowing that the phosphorylation of these proteins is not triggered by a unique soluble factor such as cytokines, hormones, or chemokines, we don’t believe that it’s possible to identify factors that are present in the serum that regulate sensitivity or resistance to TKIs. However, our data showing a heterogeneity of epigenetic alterations and of the response to JQ1 support the concept that patient-blood serum can modify the properties of the same collection of cells in a patient-dependent manner. We speculate that the composition and the magnitude of expression of diverse factors that are present in the patient-blood serum stimulate or repress multiple signaling pathways in a patient-dependent way mirroring the primary phenotype. Further exploratory studies are needed to better understand the mechanisms of cell education.

In conclusion, we present here a new approach to generate non-invasive HCC patient-derived serum educated spheroid model that can predict the clinical outcomes to TKIs based on the TICK exclusion strategy offering a highly valuable tool for drug discovery and a near perspective for personalized care of HCC patients.

## Methods

### Biological samples, cell lines, and reagents

Patient’s sera were prepared according to the protocol described elsewhere^1^. Blood samples from healthy donors were obtained from the Etablissement Français du Sang (EFS) Hauts de France – Normandie. Blood samples from HCC patients were provided by the Biological Resource Center of Montpellier University Hospital (BB-0033-00031 Montpellier), the Centre de Ressources Biologiques (CRB) Santé of Rennes (BB-0033-00056 Rennes), the Biological Resource Center of Centre Léon Bérard, Lyon, France (BB-0033-00050), and the Biological Resource Center liver biobank GH Paris-Seine-Saint-Denis (BB-0033-00027). Informed consent was obtained from all subjects and/or their legal guardian(s). The research protocol was conducted under French legal guidelines and fulfilled the requirements of the local institutional ethics committee. The study was approved by the “Direction Générale de la recherche et de l’innovation” (CODECOH, n°DC-2021-4779). This project does not involve the human person according to the legislation (article L1121-1 du code de la santé publique). Sorafenib, cabozantinib, and lenvatinib were purchased from CliniSciences (Nanterre, France).

Hepatocyte (HepG2) and monocyte (THP-1) lines were from ATCC (Molsheim, France). Hepatic stellate cell lines (TWNT-1) were from Glow Biologics (Tarrytown, NY, USA). All cell culture reagents were provided by StemCell (Saint Égrève, France). Hepatocytes, monocytes, and hepatic stellate cells were conditioned for a minimum of 2 weeks in MammoCult^®^ basal medium (StemCell) before use to sensitize them to the cell educating technology. Absence of mycoplasma contamination was verified using MycoAlert^®^ Mycoplasma Detection Kit from Lonza (Saint-Beauzire, France).

### Generation of educated spheroids and treatments

For the generation of healthy-educated spheroids used to determine the target-independent cell killing effect (TICK), we co-cultured HepG2, TWNT-1, and THP-1 cells in ultra-low attachment plates to form spheroids which then were exposed for 3 days to depleted serum from healthy donors. For HCC-educated spheroids, we co-cultured HepG2, TWNT-1, and THP-1 to form spheroids and then educated them by exposing for 3 days to depleted serum from HCC patients. We have previously shown that exposing THP-1 cells to blood serum at physiological amount triggers their differentiation into monocyte-derived macrophages and monocyte-derived dendritic cells^15^. The concentrations of the drugs used ranged from 0.01x to 240x C_max_. C_max_ of sorafenib, cabozantinib, and lenvatinib, are 20µM, 10 µM, and 0.5 µM, respectively. Educated spheroids were treated for 3 days and the cell viability was measured. Cell culture medium and cell lines sensibilization prior to educating process are described elsewhere^13^.

### Cell viability assay

Cell viability was measured using CellTiterGlo (Promega, Charbonnières-les-Bains, France) according to the manufacturer’s instructions.

### Determination of the target-independent cell killing (TICK) and therapeutic efficacy

For TICK calculation, educated healthy spheroids were treated with sorafenib, cabozantinib, or lenvatinib with concentrations up to 50x, 100x, and 240x C_max_, respectively. The cell viability was then measured after 3 days of treatment. For each drug, we generated an inhibitory dose-response curve fit with constraints (top = 100; bottom = 0) and calculated the LogIC50 and HillSlope values. The TICK was calculated using the formula:

$${\text{TICK}} = 10^{ \wedge } \left( { - \left( {\left( {{\text{Log}}\left( {\left( {100/99} \right) - 1} \right)/{\text{HillSlope}}} \right) - {\text{LogIC}}50} \right)} \right)$$

For therapeutic efficacy, HCC-educated spheroids were prepared and then treated with multikinase inhibitors for 3 days. Inhibitory dose-response curve fits were generated as above. The therapeutic efficacy for each drug and each HCC patient was determined based on the TICK value of the corresponding drug. A decrease of the cell viability before the TICK value means that the drug is efficient on this patient, and a drop of the cell viability above the TICK value means that the drug is inefficient on this patient.

### Quantitative PCR

HCC-educated spheroids were generated from a co-culture of HepG2, TWNT-1, and THP1 cell lines and then cultured for 6 days in 384 wells ultra-low attachment plates (Dutscher SAS, Bernolsheim, France). RNA extraction was performed using Arcturus^®^ PicoPure^®^ RNA Isolation Kit (Applied BiosystemsTM by Life technologiesTM) according to manufacturer’s instructions. Reverse transcription was performed using OneScript^®^ RT Mix for qPCR w/gDNAOut (Ozyme) followed by an amplification with ONEGreen^®^ FAST qPCR Premix (Ozyme) according to manufacturer’s instructions. Primers (*TIGD5*,* C8ORF33*,* NUDCD1*,* INTS8*,* ZNF623*,* STIP1*,* HSP90AB1*,* DSCC1*,* POP1*,* ARHGAP39*,* PRKDC*,* YDJC*,* PUSL1*,* EMHT2*,* DNMT1*,* EZH2*,* SUV39*,* DNMT3A*,* DNMT3B*,* UHRF1*,* SETDB1*,* BRD4*,* HDAC1*,* HDAC5*,* SIRT1*,* SIRT2*,* KDM3A*,* KDM6B*,* TET1*,* TET2*,* GusB*) were purchased from BIORAD. Quantitative PCR was performed on QuantStudio 5 Dx (Applied Biosystems by Thermo Fisher Scientific). All CTs were collected and the ∆CT were calculated by subtracting to GusB (housekeeping gene) CT. Relative Expression to GusB for each gene was calculated by using the formula RE = 2^−∆CT^.

### Graphs and statistics

Plots and statistics were generated using GraphPad Prism v9 (Dotmatics, San Diego, CA) otherwise Excel (Microsoft Office 364).

## Data Availability

Results generated in this study are not publicly available. Methods related to this study will be shared on reasonable request with permission of PredictCan Biotechnologies SAS.
